# Hidden Reservoirs of Genetic Diversity in Common Bean: Wild Populations and Local Landraces Reveal the Evolutionary Legacy of Domestication in Guerrero, Mexico

**DOI:** 10.3390/plants15213348

**Published:** 2026-10-29

**Authors:** Fernando Severiano-Galeana, Carina Gutiérrez-Flores, Xitlali Aguirre-Dugua, Alfonso Delgado-Salinas, Violeta Patiño-Conde, Jesús Padilla-Serrato, Vania Jiménez-Lobato

**Affiliations:** 1 Posgrado en Recursos Naturales y Ecología, Facultad de Ecología Marina, Universidad Autónoma de Guerrero, Acapulco 39390, Mexico; fersgal.11@gmail.com; 2 Centro de Investigaciones Biológicas del Noroeste, S.C., La Paz 23090, Mexico; caricarix@gmail.com; 3 Secretaría de Ciencia, Humanidades, Tecnología e Innovación, Ciudad de México 03940, Mexico; xitlali.aguirre@secihti.mx (X.A.-D.); jgpadillas@hotmail.com (J.P.-S.); 4 Departamento de Botánica, Instituto de Biología, Universidad Nacional Autónoma de México, Coyoacán, Ciudad de México 04510, Mexico; adelgado@ib.unam.mx; 5 Laboratorio Nacional de Análisis y Síntesis Ecológica, Escuela Nacional de Estudios Superiores Unidad Morelia, Universidad Nacional Autónoma de México, Morelia 58190, Mexico; violeta@enesmorelia.unam.mx

**Keywords:** common bean, food security, GBS, genetic diversity, Guerrero, wild relatives

## Abstract

The common bean (*Phaseolus vulgaris* L.) is a key crop for food security in Mexico, yet its genetic resources remain poorly characterized in small farmers’ agricultural fields where landraces are actively conserved. Using genotyping-by-sequencing, we assessed genetic diversity, population structure, and gene flow among wild populations (W), traditional landraces (L), and commercial varieties exchanged through local markets (M). We analyzed 73 individuals and 11,701 SNPs. Population genetic analyses revealed moderate differentiation between wild populations and cultivated forms (landraces and markets) (*Fst_L-W_* = 0.249 and *Fst_M-W_* = 0.20), whereas landraces and market varieties were weakly differentiated (*Fst_L-M_* = 0.024). Wild populations exhibited the highest genetic diversity, with the greatest expected heterozygosity (*He_W_* = 0.25) but also the highest (*Fis_W_* = 0.339). Cultivated forms showed similar expected heterozygosity between them (*He_L_* = 0.228; *He_M_* = 0.233), which was only slightly lower than that estimated in wild populations, although levels exceeded those previously reported for Guerrero and other Mexican regions. Gene flow was limited between wild and cultivated forms but high between landraces and market varieties (*Nm_L-M_* = 10.15), highlighting the role of seed exchange networks in maintaining connectivity and diversity. These findings underscore the importance of conserving wild populations, landraces, and traditional seed systems to strengthen crop resilience and food security under climate change.

## 1. Introduction

Climate change represents one of the primary threats to agriculture and biodiversity, directly affecting the productivity and stability of agricultural systems by increasing droughts, extreme temperatures, and variability in precipitation regimes. These shifts have direct consequences on global food security [1,2]. Basic crops, such as the common bean (*Phaseolus vulgaris*), are particularly sensitive to thermal and water stress, which compromises their yield and adaptive capacity under current and future climate scenarios [3]. Genetic diversity constitutes a key component of species’ adaptive capacity when facing adverse environmental conditions, as it provides the standing genetic variation upon which natural selection acts [4,5].

Within cultivated species, landraces represent reservoirs of genetic diversity. They harbor unique allelic combinations and differentiation patterns associated with local adaptation processes, which result from traditional selection and management maintained by farmers over generations [6,7,8]. Consequently, the germplasm of these varieties is not only essential for identifying climate-resilient crops and variants that are resistant to biotic and abiotic stress; it also represents a pillar of food security for peasant communities that depend on landraces for subsistence [9,10].

Wild populations belonging to the same cultivated taxon (the primary crop gene pool) are also important reservoirs of genetic diversity [11,12]. Frequently, wild populations display higher levels of genetic variation than cultivated populations. However, this pattern can be influenced by historical processes of gene flow [13], the genetic consequences of domestication (e.g., genetic bottlenecks, artificial selection, inbreeding), and management practices applied by farmers to crop populations [14,15].

Two especially significant sociocultural practices that influence the structure and composition of crop genetic diversity are the exchange of seeds between farmers and seed circulation through local markets. Local markets play an important role as interaction hubs where seeds of diverse geographical and genetic origins converge, thereby facilitating indirect gene flow across varieties and regions [16]. In Mexico, local markets have been reported to perform a fundamental role in in situ conservation by commercializing regionally produced landraces and facilitating the exchange of seeds and other agricultural products among farmers [16,17].

Previous studies on the population structure and genetic diversity of the common bean (*Phaseolus vulgaris*) show that variation is driven by the species’ predominantly autogamous reproductive system, the type of molecular marker used, diverse evolutionary and demographic processes, and hybridization or introgression events at each region, among other factors [18]. Two major gene pools of common bean have been distinguished: the Andean and the Mesoamerican. The Mesoamerican pool is more structured and genetically diverse [19,20,21,22], comprising distinct genetic groups from Brazil [23], Honduras, Guatemala, Costa Rica, Colombia [24], and Mexico. Within Mexico, distinct varieties have been identified from Oaxaca [20] and the Lerma-Santiago Basin [25], both of which exhibit high genetic diversity and are considered domestication sites in Mesoamerica, as well as varieties from Jalisco–Durango–Colima, Chiapas, and Morelos. Rodriguez et al. [24] identified a decrease in genetic diversity toward central Mexico, particularly in Guerrero, Puebla, Morelos, and the State of Mexico.

Common bean is a staple crop of considerable nutritional, economic, and social importance in Mexico. It is cultivated across approximately 1.69 million ha, with a national production of 1.29 million t and an annual per capita consumption of 11 kg. In Guerrero, bean cultivation supports approximately 27,014 producers, and the state ranks sixth nationally, producing about 11,155 t annually. Most production is conducted by smallholder farmers and relies heavily on family labor [26], emphasizing the contribution of this crop to rural livelihoods, household food security, and the conservation of native bean diversity [27]. Moreover, although national bean production in the 2024/2025 marketing year increased by 41% compared with the record-low preceding season, climatic variability continues to affect domestic supply, underscoring the strategic importance of conserving locally adapted germplasm [28].

In Guerrero landraces coexist with wild populations which may represent an important source of genetic diversity. Within wild Mesoamerican *P. vulgaris*, populations from this region have been identified as a unique lineage clearly differentiated from other wild populations [29]. Generally, higher genetic diversity has been documented in wild populations of beans compared to cultivated forms, though exceptions exist [30]. Furthermore, gene flow in beans has been found to primarily run from cultivated populations toward wild ones [31,32,33]. However, genetic diversity studies in the region remain scarce and are mostly limited to morphological and agronomic characterizations [34,35,36,37]. These uncertainties make molecular studies of beans in this region essential, as this crop lies at the base of the subsistence diet of the rural populations, and molecular data are currently lacking to establish protection and/or conservation strategies when necessary.

In this study, we therefore aimed to evaluate genetic diversity and structure through the analyses of Single-Nucleotide Polymorphisms (SNPs), as well as the magnitude and direction of gene flow, between wild and cultivated populations of *P. vulgaris* on the coast of Guerrero, Mexico. We also analyze the importance of local markets in maintaining and conserving this genetic diversity.

## 2. Results

### 2.1. Genetic Ancestry and Population Structure

Cross-entropy values decreased markedly from K = 1 to K = 5 and showed comparatively smaller reductions thereafter (K = 6 y K = 7) (Figure 1a). Although the absolute minimum cross-entropy was observed at K = 7, K = 5 was retained as the primary representation because the improvement above K = 5 was comparatively small and the additional components at K = 6 and K = 7 mainly subdivided existing ancestry patterns without corresponding to clearly differentiated groups in the PCoA or NJ tree (Figure S1). Therefore, K = 5 was interpreted as a parsimonious summary of the major ancestry patterns rather than as the unequivocal number of populations.

Across K = 3–5, wild individuals were consistently differentiated from the cultivated samples (Figure 1b–d). At K = 3, wild individuals were primarily assigned to distinct ancestry components, whereas landraces and market samples were predominantly characterized by a shared component (blue) (Figure 1b). At K = 4, an additional ancestry component revealed further differentiation among wild individuals and finer structure within the cultivated samples (Figure 1c). At K = 5, wild individuals continued to show marked ancestry differences among individuals, with most displaying relatively homogeneous ancestry profiles (Figure 1d). In contrast, landrace and market samples contained the same major ancestry components in varying proportions and frequently exhibited within-individual admixture (Figure 1d). The extensive sharing of ancestry components between landraces and market samples indicates limited genetic differentiation between these cultivated groups.

The Neighbor-Joining (NJ) tree resolved four major genetic clusters, although their bootstrap support and correspondence with the ancestry components inferred by sNMF at K = 5 varied (Figure 2). Several terminal and intermediate nodes showed high bootstrap support (>80%), whereas some of the deeper relationships among the major clusters were less strongly supported. Therefore, the NJ topology and sNMF ancestry patterns were considered partially, rather than completely, congruent.

The Core Wild cluster (green arc) included nine of the ten wild individuals, primarily representing the Wild_16, Wild_17, and Wild_36 populations, and was clearly differentiated from most cultivated samples. Internal subdivision within this cluster was broadly consistent with the heterogeneous ancestry profiles observed among wild individuals in the sNMF analysis. However, not all wild samples were included in this cluster, indicating genetic heterogeneity among the sampled wild populations.

The Colored-Seed cluster (blue arc) comprised predominantly local market accessions, including representatives of Pinto, Confeti, Ojito Escaldado, Colorado, and Amapa, together with a pair of additional black materials (Mich 32.2 and Ama 29.1). It showed heterogeneous sNMF ancestry profiles.

The Mahapan cluster (red arc) was dominated by Mahapan landraces. All samples in this cluster were collected directly from farms, although their sNMF profiles indicated contributions from multiple ancestry components. Therefore, this cluster represented a predominantly Mahapan-associated genetic assemblage rather than a completely homogeneous ancestry group.

The Widespread Cultivated Complex (orange arc) was the largest and most compositionally diverse cluster. It included predominantly the black seeds like Jamapa, Chaparro, Michigan, Negro and, Guapinol, although also includes other colored accessions such as Mantequilla, Bayo, Confeti and Ojito Escaldado as well as the Wild_35.2 sample. Several well-supported subclusters were evident within this assemblage and differed in their predominant sNMF ancestry components. Landrace and market samples were extensively interspersed throughout this cluster rather than forming provenance-specific groups, consistent with their low genetic differentiation and with the circulation of related seed materials through farms and local markets.

The position of sample Wild_35.2 may indicate genetic similarity with cultivated materials but does not, by itself, establish whether the sample reflects introgression, feralization, or crop escape. A small number of additional accessions were not assigned to any of the four major NJ clusters and instead occupied isolated or intermediate positions, some with high bootstrap support.

The Principal Coordinate Analysis (PCoA) confirm that the wild samples were genetically differentiated from the two cultivated samples. The first two axes together explained 28.4% of the total variation. The first axis accounted for the vast majority of the variance explained (18.28% of the total variance) and separated the wild samples from the remaining two bean groups. The PCoA2 axis explained a substantially smaller proportion of the variance (10.13%) and contributed slight additional separation of wild samples from the other two groups, without generating a clear separation among the cultivated samples (Landraces and Local Markets; Figure 3). The remaining 71.8% was not represented in this two-dimensional plot; therefore, the ordination provides a partial view of genetic differentiation and may not fully capture population structure.

The Analysis of Molecular Variance (AMOVA) showed that 24.38% of the total molecular variation was attributable to differences among wild, landrace, and local market groups, whereas 75.62% occurred among individuals within groups. Differentiation among the three groups was statistically significant (Φ*_ST_* = 0.244; *p* = 0.001) (999 permutations).

### 2.2. Genetic Differentiation (F_ST_), Gene Flow (Nm), and Recent Migration (M) Among Groups

The strongest genetic differentiation was observed between the wild samples and landraces (*F_ST_* = 0.249), corresponding to a restricted gene flow (*Nm* = 0.751). A similar, but slightly weaker pattern was observed between wild samples and varieties from local markets (*F_ST_* = 0.200; *Nm* = 0.998; Table 1), indicating limited genetic connectivity between wild and cultivated materials. In contrast, Landraces and Market samples exhibited low genetic differentiation (*F_ST_* = 0.024). The corresponding *Nm* value (*Nm* = 10.153), calculated from *F_ST_*, was comparatively higher than those obtained for comparisons involving wild beans.

Contemporary migration estimates obtained with BA3-SNPs indicated generally low and asymmetric migration among the three groups (Figure 4). The estimated posterior mean proportions of non-migrants (residents) ranged from 0.816 in local market samples to 0.955 in landraces. The highest recent migration proportion was estimated from landraces into local markets samples (0.171), whereas the lowest was estimated from wild beans into landraces (0.008).

### 2.3. Genetic Diversity of Common Bean

Genetic diversity estimates revealed relatively small differences in expected heterozygosity among the wild, landrace, and local market groups (Table 2). Wild samples (*n* = 10) exhibited the highest expected heterozygosity (H_E_ = 0.25), and inbreeding coefficient (*F_IS_* = 0.34), but the lowest proportion of polymorphic loci (74.5%). Landrace and local market samples showed similar expected heterozygosity (H_E_ = 0.23) which was only slightly lower than that observed in the wild group. In contrast, the proportions of polymorphic loci were higher in landraces (82.1%) and local market samples (87.6%) than in wild samples.

## 3. Discussion

To our knowledge, this study provides the first genomic characterization of wild beans, landraces, and local market varieties of *P. vulgaris* in Costa Grande, Guerrero, Mexico. Although Guerrero is one of the more biodiverse states in Mexico and harbors a high number of indigenous communities [38], the lack of molecular information limits the development of conservation and improvement strategies. Our results reveal that despite heavy anthropogenic pressure on wild habitats and strong domestication pressure on landraces, factors such as traditional agricultural management systems, ecosystem resilience, and human practices associated with marketing and germplasm exchange through local markets allow for the maintenance of moderate levels of common bean genetic diversity in these regions. These practices also drive the low genetic structure between landraces grown in farmers’ fields and market varieties. We also found higher genetic diversity in these regions than was previously reported for accessions from northern Guerrero (H_E_ < 0.15) [24], underscoring the importance of expanding evaluations into understudied regions. Conversely, the limited gene flow between wild and cultivated populations demonstrates that these two gene pools must be managed as distinct entities. This highlights the importance of conserving the natural ranges of wild populations, which harbor the highest levels of genetic diversity. It also emphasizes the need to protect and maintain local markets in the region, as they constitute an integral part of the social structure, sustaining the diversity of this crop at a regional scale.

### 3.1. Domestication and the Retention of Genetic Variability

Domestication commonly reduces genetic diversity through selection, demographic bottlenecks, founder effects, and decreases in effective population size [14,15,39]. Bitocchi et al. [19], using genomic markers in common beans, reported a significant reduction in diversity in domesticated forms relative to wild Mesoamerican and Andean populations; Schmutz et al. [40], using the common bean reference genome, noted that domestication considerably reduced nucleotide diversity and favored the fixation of genomic regions linked to agronomic traits. However, the intensity of this reduction is not uniform among crops or cultivated populations and may be moderated by their demographic history and subsequent management.

The genetic diversity patterns observed in the common bean samples of Guerrero revealed only a slight reduction in diversity in the cultivated groups—landraces and local market varieties—relative to the wild populations. Thus, although the direction of the difference was consistent with the expected genetic consequences of domestication, its magnitude was smaller than generally predicted.

A weak reduction in genetic diversity following domestication has been documented in common bean and other cultivated species. For example, Zizumbo-Villarreal et al. [30], found no evidence of reduced genetic diversity in the domesticated common bean gene pool relative to the wild gene pool in western Mexico. The authors suggested that farmer-mediated seed exchange played an important role in maintaining genetic diversity within the cultivated populations. In Mexican *Cucurbita argyrosperma*, similar levels of genetic diversity were found in wild and domesticated populations, a pattern potentially facilitated by pollinator-mediated gene flow and human seed exchange [41], whereas in cultivated pearl millet, ref. [42] found a slightly higher diversity in wild samples than in cultivated samples, attributed to direct or indirect human actions [43]. In perennial crops such as apple [44], grapevine [45], and olive [46], cultivated populations have also retained genome-wide diversity comparable to, or only slightly lower than, that of their wild relatives. These patterns have been attributed to a combination of factors such as life history traits specific to trees, human seed selection and propagation practices, breeding strategies and introgression from wild species favored by humans. Therefore, domestication does not invariably result in a pronounced genome-wide loss of genetic diversity, particularly when traditional management maintains numerous varieties and facilitates the circulation of planting material.

The relatively small difference observed between the wild and cultivated groups from Guerrero is consistent with these examples of attenuated genetic erosion. The relatively high genetic diversity retained by the cultivated groups may reflect the continued cultivation of multiple landraces, farmers’ seed-selection practices, and the exchange of seeds among households and through local markets. These processes can maintain different alleles and genotypes within the regional cultivated gene pool, partially counteracting the loss of diversity associated with domestication and selection. In particular, the close genetic similarity and high estimated gene flow between landraces and market varieties indicate that local markets are closely connected to the traditional seed system rather than representing an independent and genetically depauperate source of germplasm.

Historical introgression between wild and cultivated beans could also have contributed to the retention of ancestral variation. Nevertheless, this explanation should be considered cautiously because the moderate differentiation and restricted estimated gene flow between the wild and cultivated groups, together with the results of the recent-migration analyses, suggest that recent genetic exchange between them has been limited.

Importantly, the similar levels of diversity observed among groups do not imply an absence of domestication effects. Domestication may have altered allele frequencies, population structure, and the distribution of variation among populations without causing a pronounced reduction in overall genome-wide diversity. Accordingly, the cultivated beans of coastal Guerrero appear to retain an important proportion of the standing genetic variation found in the regional common bean gene pool, highlighting the contribution of traditional farming practices and local seed networks to the conservation of crop genetic resources.

Comparing our findings with other studies, our results indicate that varieties cultivated along the coast of Guerrero maintain higher genetic diversity than previously reported for common beans from northern Guerrero (H_E_ = 0.15) [24] and other global regions. For example, in Brazilian, for the local black and carioca beans groups, Almeida et al. [23] reported an expected heterozygosity of uH_E_ = 0.11 and uH_E_ = 0.15, respectively; in Turkey the value of H_E_ = 0.078 [47], and for Mesoamerican and Andean accessions the reported value was H_E_ = 0.168 and He = 0.102, respectively [48]. This pattern underscores the potential role of regional smallholders in maintaining genetic variability through cultivation across heterogeneous environments, informal seed exchange networks and commercialization in local markets.

Comparable dynamics have been described in other traditional diversification systems. In Ethiopia, Gelaw et al. [49], using 11,480 DArTseq SNPs, reported slightly higher mean genetic diversity in regional landraces (such as in Oromia, GD = 0.39 and PIC = 0.30) compared to global diversity (GD = 0.38), attributing this richness to cultivation across multiple environments and farmer-mediated management. In addition, Campa et al. [50] reported that traditional landraces maintained under low selection pressure by smallholder farmers harbor important recombinant blocks and preserve significant recombinant variation (~14%) that is otherwise restricted in elite cultivars, mirroring the role of coastal Guerrero farming systems in preventing regional genetic homogenization and maintaining crop diversity.

Regarding wild populations, wild beans from coastal Guerrero showed an expected heterozygosity (H_E_ = 0.25) close to that reported for wild Mesoamerican beans by Rodriguez et al. [24] (H_E_ = 0.26), although lower than the within-group gene diversity reported by Cortés et al. [21] (H_S_ = 0.30) for three wild accessions. Despite differences in sampling, these comparisons suggest that wild common bean populations from the Costa Grande of Guerrero retain genetic diversity broadly comparable to that documented in previous studies. This highlights their conservation value and supports the protection of these populations and their habitats to maintain this variation.

These findings should be interpreted in light of the scope of our sampling design. Although our sampling strategy included a broad representation of seed batches, vernacular names, farmers’ fields, and local markets, only two to three individuals were analyzed per batch or wild population. The study was designed to evaluate genetic diversity and differentiation among broad sample-origin groups rather than to quantify diversity within individual vernacular varieties. Consequently, the limited within-batch sampling may underestimate the genetic variation maintained within particular landraces or named varieties. More intensive sampling has revealed substantial diversity, admixture, and genetic subdivision within common bean varieties (e.g., Tsolakidou et al. [51]). Therefore, future studies should analyze larger numbers of individuals per batch and vernacular group to characterize within-variety variation.

### 3.2. Effects of Reproductive Isolation and Gene Flow

The genetic differentiation observed among wild, landrace, and market materials may reflect multiple, non-mutually exclusive processes. Strong wild–domesticated differentiation has previously been documented within the Mesoamerican gene pool by Rodríguez et al. [24], demonstrating that differentiation between wild and cultivated forms does not necessarily imply that they belong to different major gene pools. In our study region, differentiation may be reinforced by restricted contemporary gene flow, as wild and cultivated populations show little or no overlap in flowering phenology [34,36,52]. Farmer-mediated selection and seed-saving practices may also favor the maintenance of particular cultivated genotypes while limiting the incorporation of wild variants into cultivated germplasm [26]. Nevertheless, because gene-pool ancestry could not be independently established for all landrace and market materials, we cannot exclude the possibility that Andean ancestry or admixture contributes to some of the observed patterns. Therefore, differences among groups should be interpreted as the combined outcome of domestication and cultivation history, reproductive isolation, farmer-mediated seed management, and potentially heterogeneous genetic backgrounds, rather than as an effect of cultivation status alone.

Consistent with the observed differentiation, the detection of genetic components not observed in the cultivated samples analyzed in wild populations indicates that these populations harbor a component of genetic diversity that is not represented in the cultivated materials analyzed here. Nevertheless, the relatively small wild-bean sample limits the representativeness of these findings, which should not be generalized to all wild populations in Guerrero. Despite this limitation, conserving the sampled wild populations remains important for preserving distinct genetic components and maintaining the regional genetic diversity of *P. vulgaris*.

### 3.3. Local Markets as Bridges for Genetic Exchange

The heterogeneous or admixed sNMF profiles, together with their distribution in the PCoA, suggest that the four broad NJ clusters summarize the predominant genetic relationships but do not capture the full extent of substructure within the sampled germplasm. Overall, the PCoA, NJ, and sNMF analyses consistently distinguished a principal wild-associated genetic component while showing limited separation between landrace and market samples. Nevertheless, the four NJ clusters did not correspond one-to-one with the five ancestry components inferred by sNMF, indicating finer-scale genetic heterogeneity and admixture within the major tree-based clusters.

The low genetic differentiation between landraces and varieties commercialized in local markets, was consistent with high gene flow. Nevertheless, the *Nm* value should be interpreted cautiously. Its calculation assumes migration–drift equilibrium, equal population sizes, neutrality, and random mating, assumptions that are unlikely to be fully met in a predominantly self-pollinating domesticated crop subject to artificial selection. Thus, this value represents only an indirect historical approximation under an idealized island model and should not be interpreted as a direct estimate of contemporary migrants per generation.

Considering this, the result suggests that local commercial networks, such as markets, function as vital bridges for genetic exchange between agricultural communities. In this sense, markets represent more than just economic spaces; they act as dynamic mechanisms for the dispersal and redistribution of regional agricultural genetic diversity [16,53]. Llamas-Guzmán et al. [17] noted that informal seed exchange among farmers and local markets in the municipalities of Ixtenco and Huamantla, in the state of Tlaxcala, Mexico, contributes significantly to maintaining and conserving agrobiodiversity. Similarly, Severiano-Galeana et al. [26] reported that 21% of farmers interviewed on the coast of Guerrero acquire their seeds for planting or consumption from local shops and markets, while 79% engage in seed exchanges with relatives and neighbors from surrounding communities. Consequently, seeds commercialized in local markets tend to act as genetic mosaics derived from multiple sources, reducing genetic differentiation across cultivated populations. Thus, local markets function as in situ conservation hubs by promoting the exchange of local seeds among regional farmers and selling varieties produced directly in the field.

In contrast, we detected only very faint signals of recent migration from market and landraces varieties toward wild populations. This could be due to occasional crop-to-wild introgression events in areas where cultivated fields are close to wild populations, likely favored by anthropogenic disturbances or crop escapes. Several studies have documented bidirectional gene flow events within wild–weedy–domesticated complexes, particularly in regions where both reservoirs coexist in intricate agroecological mosaics [54]. This gene flow can threaten the survival of crops’ wild relatives through the introgression of maladaptive genes that could lead to local extinction or turn wild populations into aggressive weeds [55,56]. However, in this study, we detected minimal gene flow, indicating clear genetic independence between the two reservoirs.

### 3.4. Inbreeding and Heterozygosity Patterns Among Sampling Groups

A relatively high inbreeding coefficient (*F_IS_* = 0.34) was observed in the pooled wild-bean group, which also exhibited the highest expected heterozygosity (*H_E_* = 0.25). Although this pattern may appear paradoxical, some evolutionary and demographic processes can explain the coexistence of high population-level genetic diversity alongside high individual-level homozygosity. One primary explanation is the reproductive biology of the species. Wild *P. vulgaris* has a high self-fertilization rate, which increases inbreeding [57]. In natural environments, wild bean vines grow isolated among trees and shrubs, promoting high rates of self-pollination or mating between closely related individuals, growing close to their maternal plant [58], which increases homozygosity and drives high inbreeding values despite high regional genetic diversity.

Another possible explanation relates to the small size and partial isolation of the wild populations analyzed. In small populations, genetic drift can rapidly increase homozygosity and favor mating among related individuals, particularly when external gene flow is limited [59,60]. Likewise, a small effective population size can accelerate the random loss of alleles and increase the fixation of deleterious variants, intensifying inbreeding levels [61].

On the other hand, the negative *Fis* observed in landraces and market samples indicated an excess of heterozygosity relative to Hardy–Weinberg expectations. These groups comprise multiple varieties and maternal seed lots collected across localities and therefore should not be regarded as a single panmictic population. Its *Fis* estimate must consequently be interpreted cautiously. The residual excess may reflect occasional outcrossing or seed-mediated admixture among locally maintained materials.

Taken together, our results demonstrate that the coastal region of Guerrero constitutes an important reservoir of ancestral genetic diversity for the common bean, preserved in both wild populations and landraces. This underscores the need to implement comprehensive in situ conservation strategies and sustainable utilization plans for this germplasm that balance the protection of wild populations with the dynamic maintenance of landraces managed by farmers. Furthermore, this study highlights the fundamental role of farmers and local markets as key actors in the conservation, circulation, and maintenance of regional genetic diversity. Through seed exchange, selection, and commercialization, these sociocultural systems actively contribute to preserving unique genetic lineages and generating continuous variation. In this context, local markets function not only as commercial venues but also as central hubs for regional germplasm conservation and genetic connectivity. The genetic diversity and ancestral lineages identified here represent a valuable and potential source of variation and constitute the essential raw material needed to strengthen the resilience and agronomic adaptation of *Phaseolus vulgaris* when facing the challenges imposed by global climate change.

## 4. Materials and Methods

### 4.1. Study Area and Sample Collection

Seeds of *P. vulgaris* were obtained from four municipalities in the coastal region of Guerrero, Mexico: Atoyac de Álvarez, Coyuca de Benítez, Tecpan de Galeana, and Tecoanapa (Figure 5; Table S1). This region is the primary producer of common bean in the state [62]. The study area has a warm, subhumid climate, with an average annual temperature of 28.5 °C and annual precipitation ranging from 800 to 2000 mm [63]. Samples were categorized into three groups according to their origin: (a) landraces grown in farmers’ agricultural plots (designated as L, *n* = 21 batches); (b) varieties from local markets distributed across the four municipalities (M, *n* = 8 batches); and (c) wild populations, collected from four different populations (W, *n* = 4 populations).

Four geographically distinct wild bean populations, separated from one another by several kilometers, were initially identified during the flowering period. Individual plants within each population were marked, and the sites were revisited during pod maturation. Seeds were collected separately from the previously marked plants, with each wild seed lot corresponding to a distinct maternal plant.

Vernacular names of all cultivated samples (landraces and markets) were recorded, producing a list of 15 names of cultivated varieties as recognized by local farmers and shop owners.

### 4.2. Greenhouse Plant Germination

Several seeds from each cultivated batch (landrace and market) and each wild maternal seed lot were germinated under greenhouse conditions at the Escuela Nacional de Estudios Superiores (ENES), Unidad Morelia of the National Autonomous University of Mexico (UNAM). Once the seedlings had developed at least three true leaves, leaf tissue was collected and immediately transported to the laboratory for DNA extraction. Two to three plants were selected from each cultivated batch, whereas one plant was selected from each of three maternal seed lots collected within each wild population. Before quality filtering, the resulting dataset comprised 81 DNA samples: 12 wild individuals, 45 landrace individuals, and 24 market individuals.

### 4.3. DNA Extraction

DNA was extracted at the Genomics Laboratory of the ENES, Unidad Morelia, UNAM. For individual DNA extractions, 30 mg of fresh leaf tissue was ground using liquid N_2_ and processed with the DNeasy Mini Plant Kit (QIAGEN GmbH, Hilden, Germany), following the manufacturer’s instructions. DNA integrity was verified via 1% agarose gel electrophoresis using a high-molecular-weight lambda ladder as a reference. Additionally, DNA quality was determined by measuring spectrophotometric absorbance ratios on a Eppendorf BioSpectrometer^®^ basic (Eppendorf SE, Hamburg, Germany) to ensure appropriate 260/280 nm and 260/230 nm ratios to rule out contamination by proteins and carbohydrates, respectively. DNA concentration was measured using a Qubit^®^ 2.0 Fluorometer (Invitrogen, Carlsbad, CA, USA). and homogenized to a concentration of 50 ng/μL.

### 4.4. Genome Sequencing

A total of 81 individuals, 12 from wild populations, 45 from landraces collected from farmer’s fields, and 24 from local markets, were sequenced. Genotyping-by-sequencing (GBS) was performed via paired-end sequencing with a target fragment selection size of 150 bp and an average read depth of 10X. Library preparation and genome sequencing were carried out by SNPSaurus LLC (Eugene, OR, USA; https://www.snpsaurus.com/).

### 4.5. SNP Identification and Variant Calling

Sequence quality was analyzed using FastQC v0.12.1 to identify adapters, low-quality reads, or regions with inconsistent quality. Reads were mapped and aligned to the common bean reference genome (PhaVulg1_0: GCA_000499845.1) [40] using the Burrows–Wheeler Aligner (BWA) [64] algorithm. The resulting alignment files in SAM (Sequence Alignment Map) format were converted to binary format (BAM) and sorted using SAMtools v1.13 [65]. Finally, duplicate reads resulting from library preparation were removed using GATK4 v4.6.1.0 [66].

SNP calling was performed using the HaplotypeCaller module, and individual samples were joint-called into a single VCF file via CombineGVCFs, both of which are tools within the Genome Analysis Toolkit software (Gatk v.4.6.1.0) [66]. The final genotyped VCF file was generated using GenotypeGVCFs v4.6.1.0. A total of 23,699,568 SNPs were initially obtained and subsequently filtered. Variants were retained according to the following criteria: a Phred-scaled quality score ≥30, a minor allele frequency (MAF) ≥ 0.05, biallelic status, and no more than 20% missing data per site. Potential paralogous loci were identified using HDplot following McKinney et al. [67]. This approach combines the proportion of heterozygous individuals at each locus (H) with the deviation of allelic read depth from the expected 1:1 ratio in heterozygous genotypes (D, expressed as a z-score). HDplot was applied to the complete dataset and separately to the wild (*n* = 10), landrace (*n* = 41), and local-market (*n* = 22) groups to detect potential group-specific mapping artifacts. SNPs with H > 0.60 and −7 < D < 7 were classified as potential paralogous loci. SNPs identified as potential paralogs in at least one of the global or group-specific analyses were removed. After paralog filtering, linkage disequilibrium (LD) pruning was performed using PLINK v1.9 with a sliding window of 50 SNPs, a step size of five SNPs, and an ***r***^2^ threshold of 0.5 [68]. After LD pruning, the final dataset comprised 11,701 SNPs and 73 individuals: 10 from wild populations, 41 landraces collected from farmers’ fields, and 22 samples obtained from local markets. This dataset was used for all subsequent downstream analyses. All bioinformatic analyses were executed on the high-performance computing cluster at the Laboratorio Nacional de Análisis y Síntesis Ecológica (LANASE), ENES Unidad Morelia, UNAM.

### 4.6. Data Analysis

#### 4.6.1. Genetic Ancestry and Population Structure

To determine the genetic structure among the three provenance groups, individual ancestry coefficients were calculated using Sparse Non-negative Matrix Factorization (sNMF), which utilizes sparse least-squares optimization algorithms to infer population structures [69]. The analysis evaluated a range of clusters from K = 1 to K = 7, with 10 independent replicates per K value. The optimal number of clusters was determined using the cross-entropy criterion to select the most probable number of genetic groups, facilitated by the LEA v3.20 package in RStudio [70,71]. Unlike other Bayesian clustering models such as STRUCTURE, sNMF relaxes the assumption of the Hardy–Weinberg equilibrium, making it highly suitable for large SNP datasets [70].

Additionally, a Neighbor-Joining (NJ) phylogenetic tree was constructed with 1000 bootstrap replicates using MEGA 12.1 software [72]. A Principal Coordinate Analysis (PCoA) was also performed to explore patterns of similarity or dissimilarity among batches based on a Nei’s genetic distance matrix, using RStudio v4.5.2 [73]. To determine the relative contribution of each ancestral lineage within the provenance groups, mean ancestry coefficients were calculated at the group level from the Q-matrix using RStudio v4.5.2 [73].

An Analysis of Molecular Variance (AMOVA) was performed to partition molecular variation between the three biological groups (wild, landrace, and market) and among individuals within groups. The analysis was conducted with the poppr.amova function in the R package poppr [74,75], which implements the AMOVA procedure available in ade4 [76]. Differentiation among groups was quantified using the Φ*_ST_* statistic, and its statistical significance was evaluated with 999 permutations using the randtest function in ade4.

#### 4.6.2. Genome Wide Genetic Diversity

Genetic diversity analyses were conducted across the three provenance groups. The following diversity parameters were estimated using the hierfstat package in R version 4.6.1 [73]: expected heterozygosity (H_E_), observed heterozygosity (H_O_), number of polymorphic loci (*p*), percentage of polymorphic loci (*%p*) and, local inbreeding coefficient (*F_IS_*) [77].

#### 4.6.3. Gene Flow and Migration Rates

To characterize genetic connectivity among the three provenance groups, pairwise *Fst* values were first estimated according to Weir and Cockerham using the hierfstat package in R [77]. Historical gene flow (*Nm*) was then indirectly approximated from each pairwise *Fst* estimate, following the assumptions of the island model [78]. Here, *Nm* represents an indirect measure of historical genetic connectivity rather than a direct estimate of the number of migrants.

Contemporary proportions of recent migration (m) among groups were estimated independently using BA3-SNPs v3.0.4 [79]. These estimates represent migration occurring during the last two generations. Ten independent runs were performed, each consisting of 3,000,000 Markov chain Monte Carlo (MCMC) iterations, with the first 1,000,000 iterations discarded as burn-in and parameters sampled every 73 iterations. The mixing parameters were set to the default BA3 values [79].

## Figures and Tables

**Figure 1:**
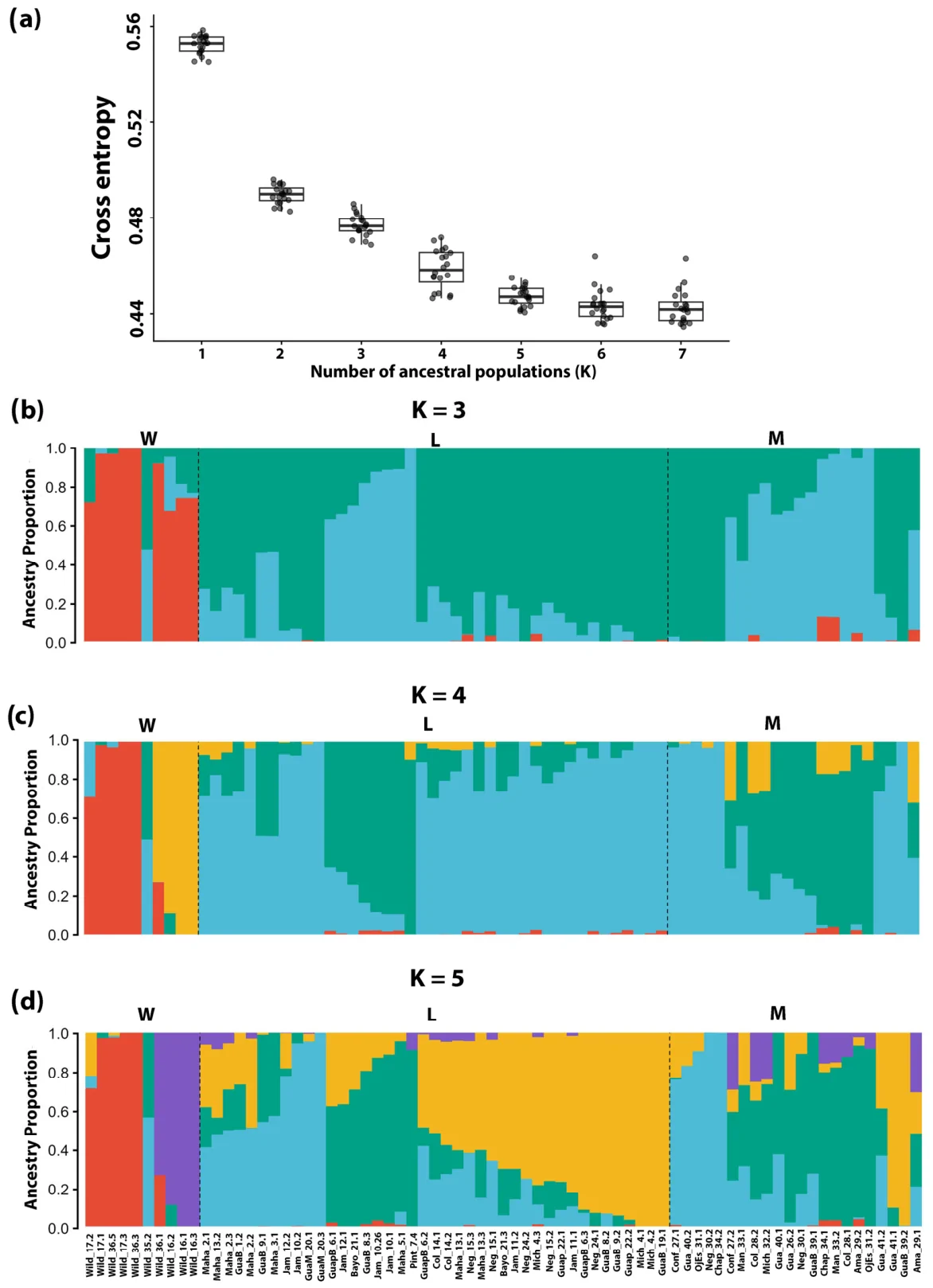
Population genetic structure of *Phaseolus vulgaris* inferred with Sparse Non-negative Matrix Factorization, sNMF. (**a**) Cross-entropy values obtained using sNMF method for different numbers of genetic clusters (K). (**b**) Ancestry coefficients for K = 3. (**c**) Ancestry coefficients for K = 4. (**d**) Ancestry coefficients for K = 5. Origin group: W: wild populations; L: local landraces; M: local market varieties.

**Figure 2:**
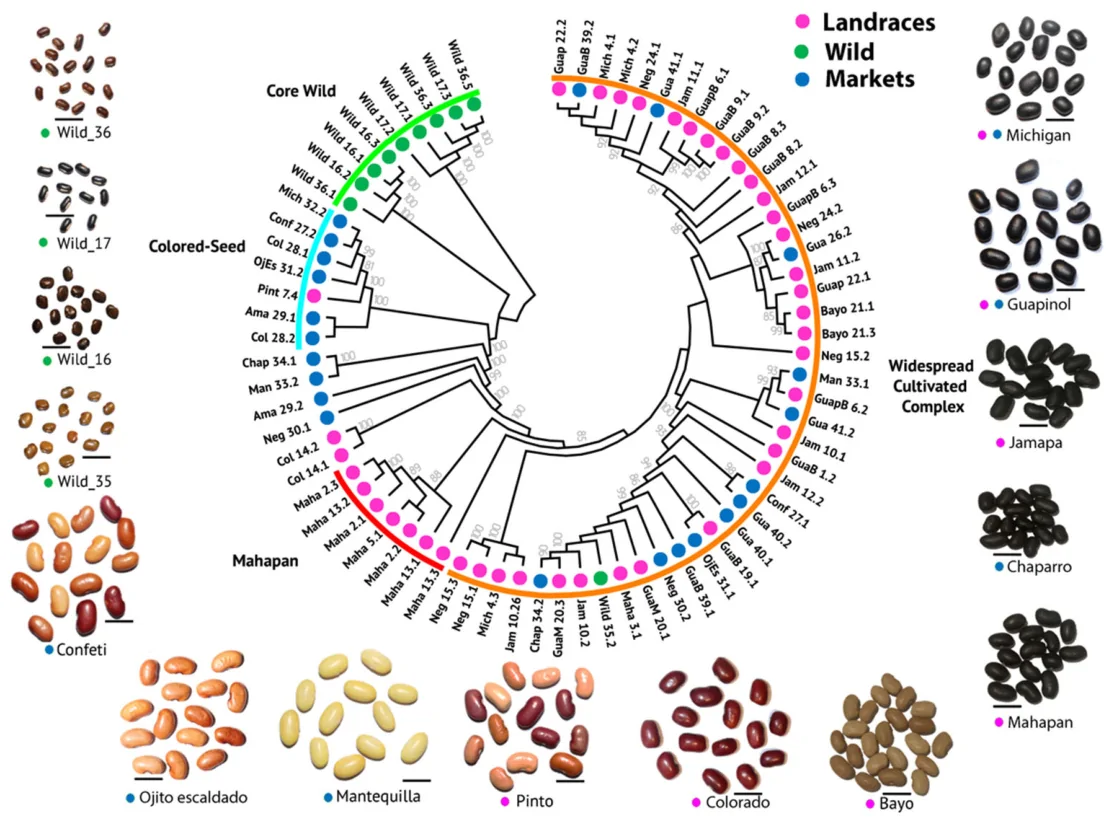
Neighbor-Joining (NJ) phylogenetic tree based on 11,701 genome-wide SNPs of *Phaseolus vulgaris*. Numerical values at nodes indicate bootstrap support obtained from 1000 replicates. Colored circles at the tips represent sampling origin groups analyzed. Colored outer arcs delimit the four major NJ clusters. Accessions not covered by an outer arc were not assigned to these four broad clusters because they occupied isolated or intermediate positions in the tree. Name of varieties: Ama: Amapa; Bayo: Bayo; Chap: Chaparro; Col: Colorado; Conf: Confeti; Guap: Guapinol; GuapB: White Guapinol; GuapM: Purple Guapinol; Jam: Jamapa; Maha: Mahapan; Man: Mantequilla; Mich: Michigan; Neg: Black; OjEs: Ojito Escaldado; Pint: Pinto.

**Figure 3:**
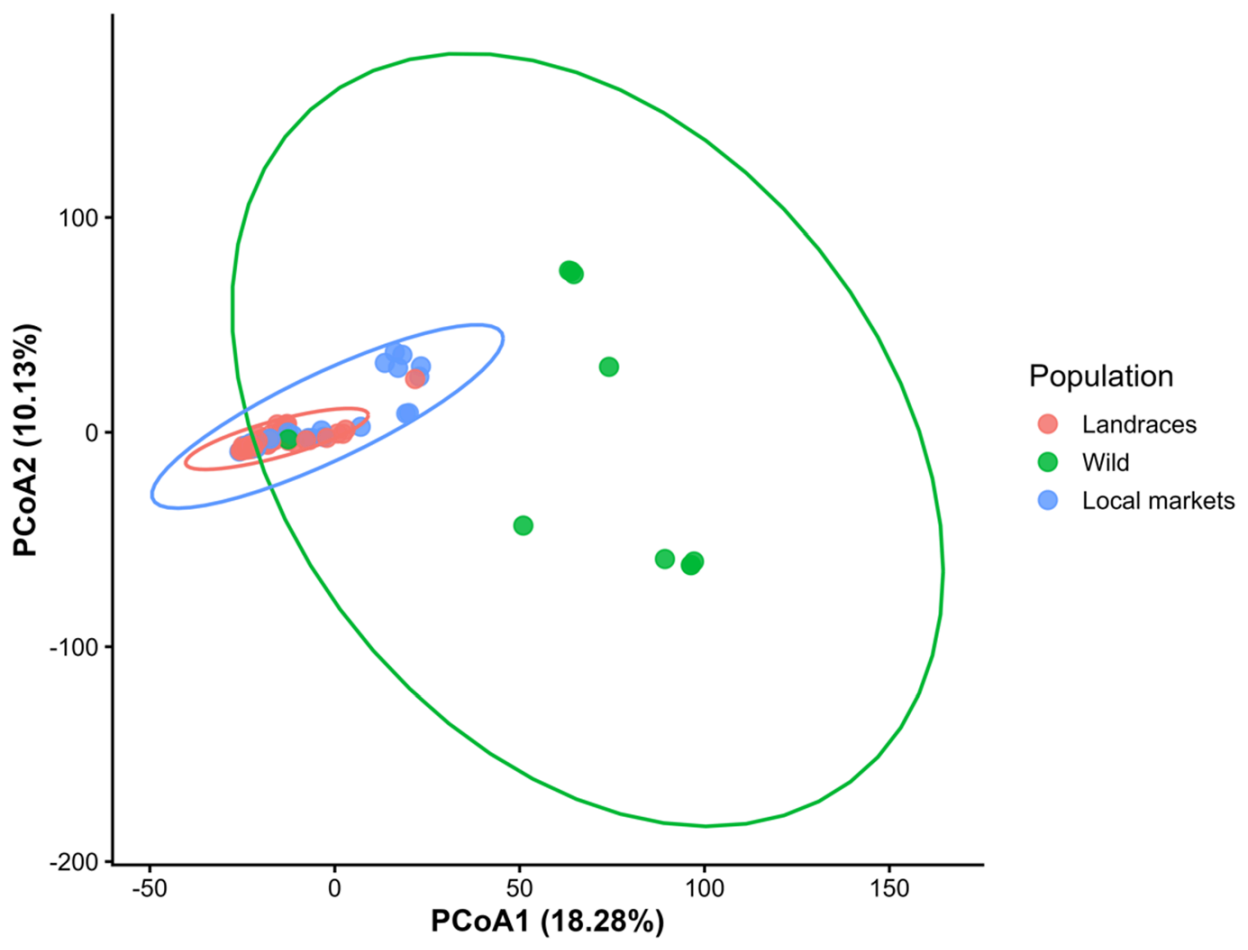
Principal Coordinates Analysis (PCoA) of 73 *Phaseolus vulgaris* accessions. Each point represents an individual, and colors indicate the origin groups analyzed.

**Figure 4:**
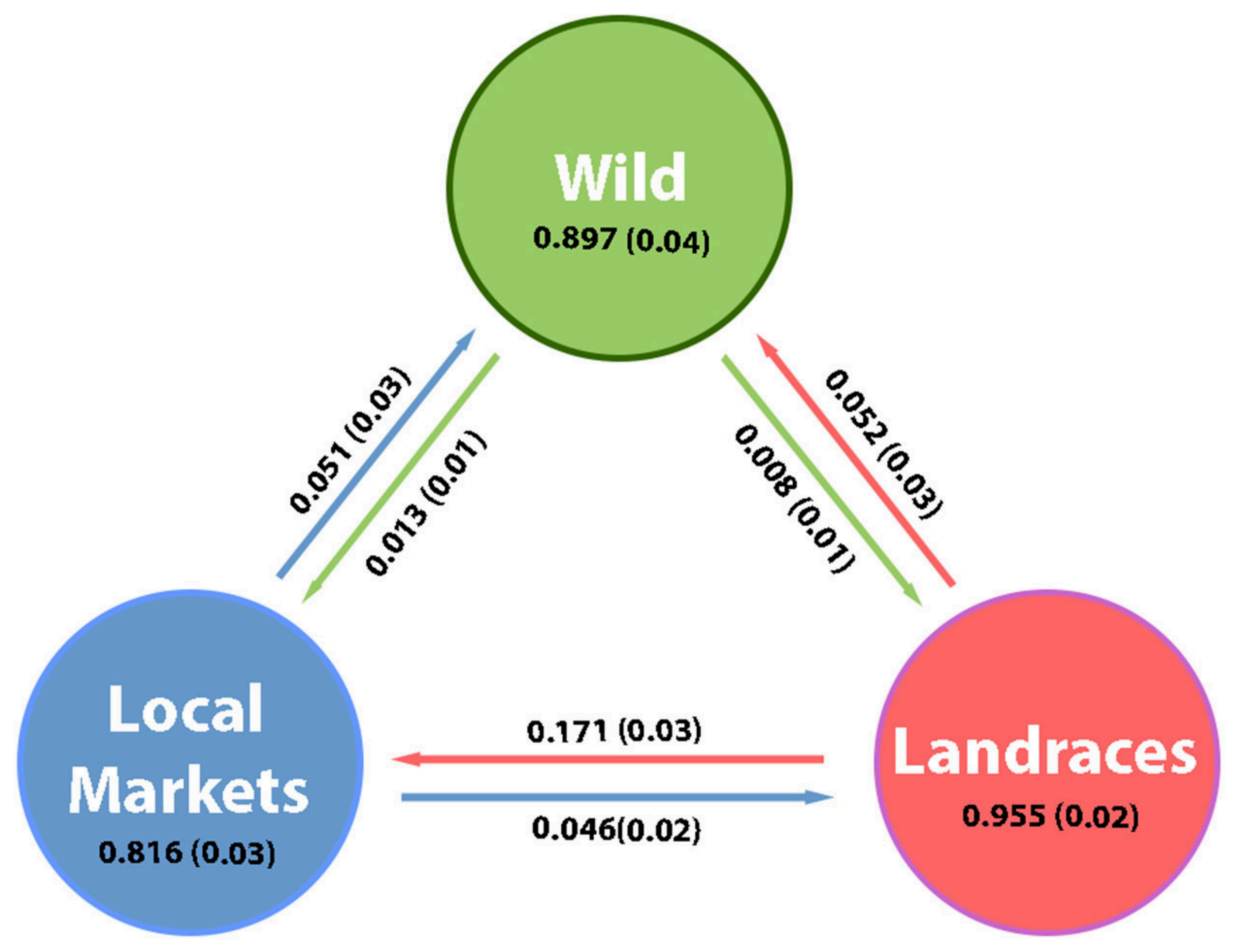
Recent migration rates in the last two generations (standard deviation) among common bean (*Phaseolus vulgaris*) groups in the coast of Guerrero, Mexico. The arrows represent the direction of the estimated proportions of recent migrants between pairs of populations. Values inside the circles represent the posterior mean proportion of nonmigrants (residents) within each group.

**Figure 5:**
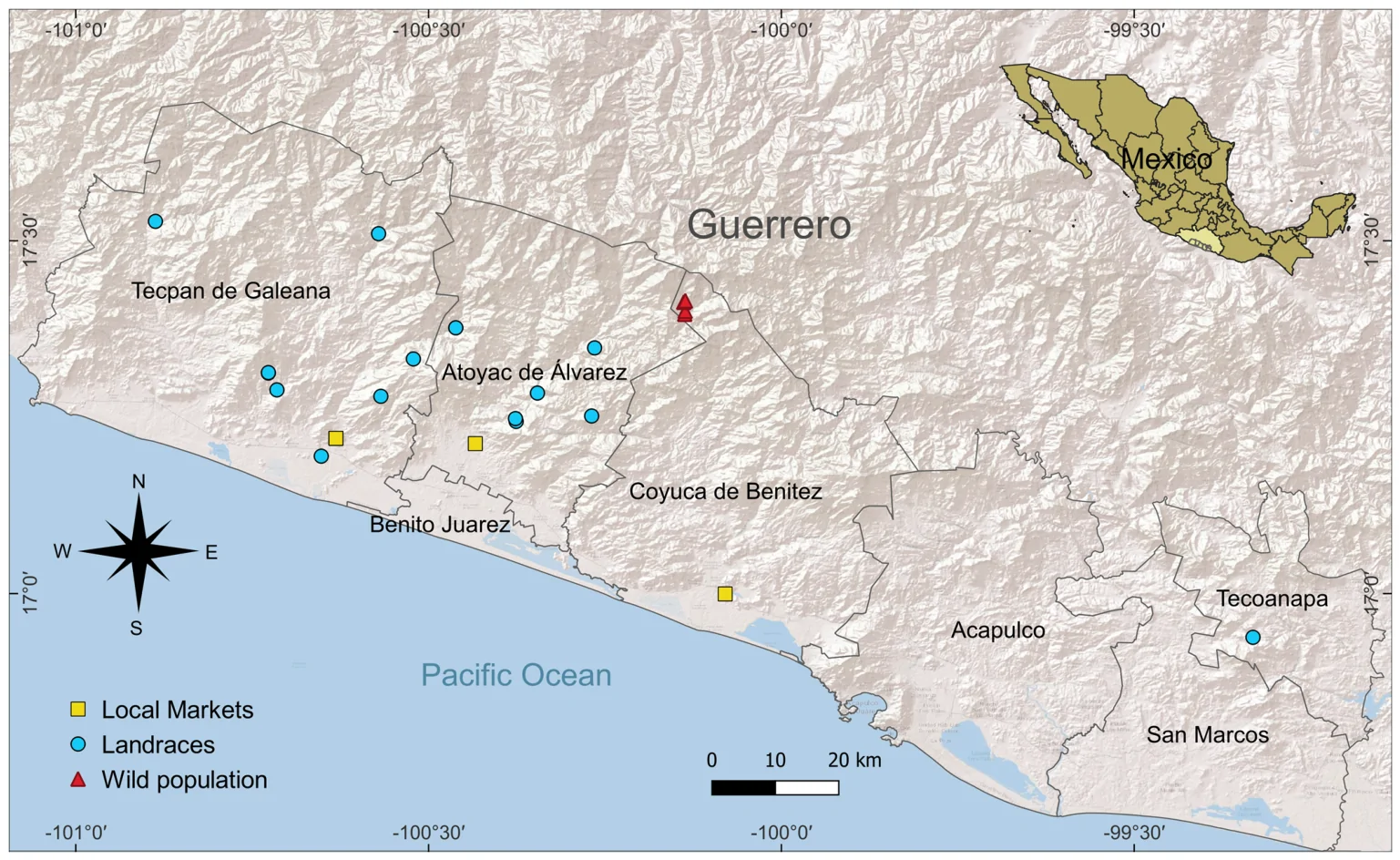
Study area and sampling sites of common bean (*Phaseolus vulgaris*) seeds collected along the coast of Guerrero, Mexico.

**Table 1:** Pairwise genetic differentiation (*F_ST_*) (lower diagonal) and gene flow (*Nm*) (upper diagonal) based on SNP markers among three common bean (*Phaseolus vulgaris*) groups in the coast of Guerrero, Mexico.

*F_ST_/Nm*	Wild	Landraces	Local Markets
Wild	---	0.751	0.998
Landraces	0.249	---	10.153
Local markets	0.200	0.024	---

**Table 2:** Genome-wide genetic diversity estimates in three common bean (*Phaseolus vulgaris*) groups in the coast of Guerrero, Mexico. Standard deviations are shown in parentheses.

Groups	*n*	*p*	%*p*	H_O_ (s.d)	H_E_ (s.d)	F_IS_
Wild	10	8717	74.50	0.165 (0.19)	0.250 (0.20)	0.339
Landraces	41	9601	82.05	0.273 (0.28)	0.228 (0.11)	−0.193
Local markets	22	10,254	87.63	0.229 (0.23)	0.234 (0.15)	−0.019
Total	73		100%			

*n*: number of samples per group; *p*: number of polymorphic loci; %*p*: percentage of polymorphic loci; H_O_: observed heterozygosity; H_E_: expected heterozygosity; F_IS_: inbreeding coefficient.

## Data Availability

The VCF dataset is undergoing additional analyses beyond the scope of this manuscript. We would therefore like to state that the data supporting the findings reported here are available from the corresponding author upon reasonable request.

## Supplementary Materials

The following supporting information can be downloaded at: https://www.mdpi.com/article/10.3390/plants15213348/s1. Figure S1. Population genetic structure of *Phaseolus vulgaris* inferred with Sparse Non-negative Matrix Factorization, sNMF. (a) Ancestry coefficients for K = 6; (b) Ancestry coefficients for K = 7. Origin group: W: wild populations; L: local landraces; M: Local market varieties. Table S1. Common bean (*Phaseolus vulgaris*) samples collected in the Costa Grande and Costa Chica regions of Guerrero, Mexico.
