# Supplementary material for: Hidden Reservoirs of Genetic Diversity in Common Bean: Wild Populations and Local Landraces Reveal the Evolutionary Legacy of Domestication in Guerrero, Mexico
Source: Plants (Basel). 2026 Oct 29;15(21):3348. doi: 10.3390/plants15213348 (PMC13766129; doi:10.3390/plants15213348)
Supplement: Supplementary file 1 [file plants-15-03348-s001.zip › plants-4525239-supplementary.pdf]

Supplementary material

Table S1. Common bean (*Phaseolus vulgaris*) samples collected in the Costa Grande and Costa Chica regions of Guerrero, Mexico.

Municipality	Origin group	Varieties	Latitude	Longitude	Altitude	Vegetation type
AA	Landraces	Mahapan purple	17°14'38.39''N	100°22'33.6''W	630 m a.s.l	Tropical dry forest
AA	Landraces	Mahapan purple	17°14'13.19''N	100°22'22.8''W	685 m a.s.l	Tropical dry forest
AA	Landraces	Mahapan purple	17°14'52.8''N	100°22'37.19''W	528 m a.s.l	Tropical dry forest
AA	Landraces	Jamapa	17°17'03.0''N	100°20'45.0''W	524 m a.s.l	Tropical dry forest
AA	Landraces	Jamapa	17°17'03.0''N	100°20'45.0''W	524 m a.s.l	Tropical dry forest
AA	Landraces	Jamapa	17°17'03.0''N	100°20'45.0''W	524 m a.s.l	Tropical dry forest
AA	Landraces	Mahapan purple	17°15'07.6''N	100°16'08.4''W	1004 m a.s.l	Pine–oak forest
AA	Landraces	Guapinol purple	17°22'35.92''N	100°27'41.63''W	470 m a.s.l	Tropical dry forest
AA	Landraces	Bayo	17°22'35.92''N	100°27'41.63''W	470 m a.s.l	Tropical dry forest
AA	Landraces	Black	17°17'03.0''N	100°20'45.0''W	524 m a.s.l	Tropical dry forest
TG	Landraces	Guapinol white	17°30'17.99''N	100°53'13.99''W	592 m a.s.l	Tropical dry forest
TG	Landraces	Michigan	17°30'36''N	100°34'15.59''W	622 m a.s.l	Tropical dry forest
TG	Landraces	Guapinol White	17°18'45.05''N	100°43'37.86''W	519 m a.s.l	Tropical dry forest
TG	Landraces	Pinto	17°19'57''N	100°31'18''W	706 m a.s.l	Tropical dry forest
TG	Landraces	Guapinol White	17°17'19.06''N	100°42'54.18''W	370 m a.s.l	Tropical dry forest
TG	Landraces	Guapinol White	17°18'47.4''N	100°43'37.86''W	650 m a.s.l	Tropical dry forest
TG	Landraces	Guapinol	17°11'41''N	100°39'08''W	17 m a.s.l	Tropical dry forest
TG	Landraces	Guapinol	17°16'46.44''N	100°34'03.98''W	700 m a.s.l	Tropical dry forest
Teco	Landraces	Colorado	16°56'15''N	99°19'54''W	798 m a.s.l	Pine–oak forest

Teco	Landraces	Black	16°56'26''N	99°19'48''W	798 m a.s.l	Pine–oak forest
AA	Local markets	Guapinol	17°12'45"N	100°26'02"W	NA	NA
AA	Local markets	Colorado	17°12'45"N	100°26'02"W	NA	NA
CB	Local markets	Black	16°59'58"N	100°04'47"W	NA	NA
CB	Local markets	Chaparro	16°59'58"N	100°04'47"W	NA	NA
TG	Local markets	Michigan	17°13'12"N	100°37'53"W	NA	NA
TG	Local markets	Guapinol White	17°13'12"N	100°37'53"W	NA	NA
TG	Local markets	Guapinol	17°13'12"N	100°37'53"W	NA	NA
TG	Local markets	Guapinol	17°13'12"N	100°37'53"W	NA	NA
AA	Wild	Pob_1	17°23'45.5"N	100°08'13.6"W	1369 m a.s.l	Pine–oak forest
AA	Wild	Pob_2	17°24'01.0"N	100°08'11.3"W	1404 m a.s.l	Pine–oak forest
AA	Wild	Pob_3	17°24'46.7"N	100°08'18.4"W	1706 m a.s.l	Pine–oak forest
AA	Wild	Pob_4	17°24'53.7"N	100°08'10.6"W	1752 m a.s.l	Pine–oak forest

TG: Tecpan de Galeana; AA: Atoyac de Álvarez; CB: Coyuca de Benítez; Teco: Tecoanapa.

Figure S1

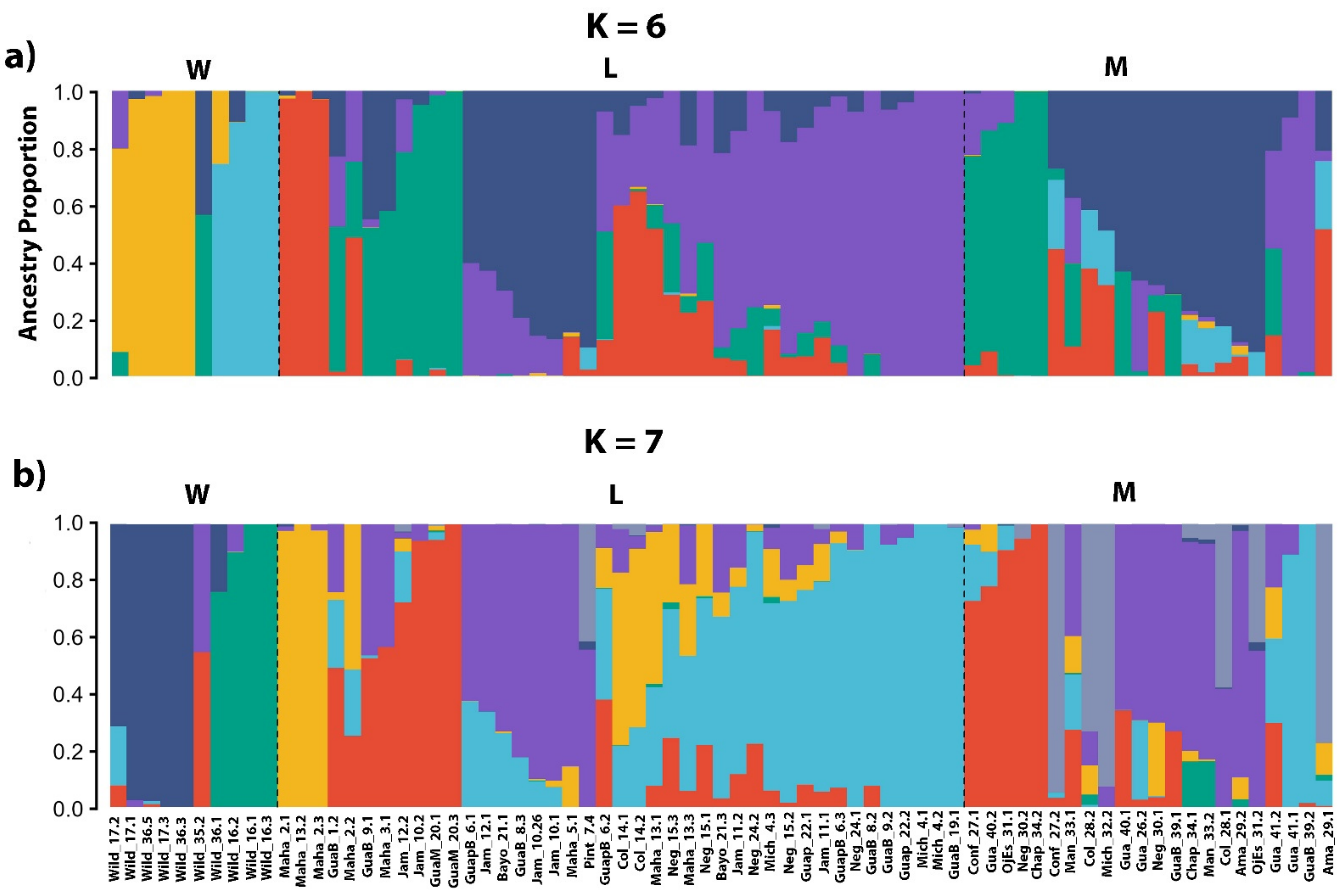

Figure S1. Population genetic structure of *Phaseolus vulgaris* inferred with Sparse Non-negative Matrix Factorization, sNMF. a) Ancestry coefficients for K = 6; b) Ancestry coefficients for K = 7. Origin group: W: wild populations; L: local landraces; M: Local market varieties.
